# Diet-induced hypercholesterolemia promotes androgen-independent prostate cancer metastasis via IQGAP1 and caveolin-1

**DOI:** 10.18632/oncotarget.3476

**Published:** 2015-03-02

**Authors:** Hyeongsun Moon, Jayde E. Ruelcke, Eunju Choi, Laura J. Sharpe, Zeyad D. Nassar, Helle Bielefeldt-Ohmann, Marie-Odile Parat, Anup Shah, Mathias Francois, Kerry L. Inder, Andrew J. Brown, Pamela J. Russell, Robert G. Parton, Michelle M. Hill

**Affiliations:** ^1^ The University of Queensland Diamantina Institute, The University of Queensland, Translational Research Institute, Brisbane, QLD, Australia; ^2^ School of Veterinary Science, The University of Queensland, Gatton, QLD, Australia; ^3^ School of Biotechnology and Biomolecular Sciences, The University of New South Wales, Sydney, NSW, Australia; ^4^ The School of Pharmacy, The University of Queensland, Pharmacy Australia Centre of Excellence, Woolloongabba, QLD, Australia; ^5^ Institute for Molecular Bioscience, The University of Queensland, Brisbane, QLD, Australia; ^6^ Australian Prostate Cancer Research Centre–Queensland and Institute of Health and Biomedical Innovation, Faculty of Health, Queensland University of Technology, Translational Research Institute, Brisbane, QLD, Australia; ^7^ Current address: Department of Biomedical Sciences, College of Veterinary Medicine, Cornell University, Ithaca, NY, USA

**Keywords:** Prostate cancer, hypercholesterolemia, metastasis, IQGAP1, caveolin-1

## Abstract

Obesity and metabolic syndrome are associated with several cancers, however, the molecular mechanisms remain to be fully elucidated. Recent studies suggest that hypercholesterolemia increases intratumoral androgen signaling in prostate cancer, but it is unclear whether androgen-independent mechanisms also exist. Since hypercholesterolemia is associated with advanced, castrate-resistant prostate cancer, in this study, we aimed to determine whether and how hypercholesterolemia affects prostate cancer progression in the absence of androgen signaling. We demonstrate that diet-induced hypercholesterolemia promotes orthotopic xenograft PC-3 cell metastasis, concomitant with elevated expression of caveolin-1 and IQGAP1 in xenograft tumor tissues. *In vitro* cholesterol treatment of PC-3 cells stimulated migration and increased IQGAP1 and caveolin-1 protein level and localization to a detergent-resistant fraction. Down-regulation of caveolin-1 or IQGAP1 in PC-3 cells reduced migration and invasion *in vitro*, and hypercholesterolemia-induced metastasis *in vivo*. Double knock-down of caveolin-1 and IQGAP1 showed no additive effect, suggesting that caveolin-1 and IQGAP1 act *via* the same pathway. Taken together, our data show that hypercholesterolemia promotes prostate cancer metastasis independent of the androgen pathway, in part by increasing IQGAP1 and caveolin-1. These results have broader implications for managing metastasis of cancers in general as IQGAP1 and hypercholesterolemia are implicated in the progression of several cancers.

## INTRODUCTION

Epidemiological studies have long reported an association between hypercholesterolemia, obesity and several types of cancers, with cholesterol-lowering drugs showing clinical benefit in the prevention and delay of prostate cancer progression [1, 2]. Recent studies have begun to reveal the potential mechanisms by which the cholesterol biosynthetic pathway and its metabolites impact carcinogenesis [3-6]. Elevated blood cholesterol levels may enhance the androgen receptor pathway *via* intratumoral *de novo* androgen synthesis in the prostate, contributing to prostate cancer progression [7]. The cholesterol metabolite 27-hydroxycholesterol increases estrogen receptor function in breast cancer [4, 5], while oxidized cholesterol metabolites (oxysterols) released from tumor cells promote angiogenesis and immunosuppression in the tumor microenvironment [8].

In addition to its role in steroid hormone synthesis and function, cholesterol is an essential structural component of mammalian membranes. Cholesterol-enriched membrane microdomains, also termed lipid rafts, are reported to regulate a myriad of cellular functions [9]. Previous studies suggest a potential role for membrane cholesterol [10]. and the cholesterol-binding membrane protein, caveolin-1, in promoting prostate cancer progression [11]. Elevation of lipid profiles, including cholesterol, is a common side effect of androgen deprivation therapy for advanced prostate cancer [12]. Given the association of hypercholesterolemia with advanced, castrate-resistant prostate cancer which is insensitive to androgen pathway blockade [13], it is important to determine whether high circulating and cellular cholesterol can directly affect advanced prostate cancer progression in the absence of androgen signaling.

In this study, we examined the effects of diet-induced hypercholesterolemia on androgen-receptor negative prostate cancer progression, and investigated the potential mechanisms. We show that diet-induced hypercholesterolemia accelerated secondary tumor metastases to lymph node, lung and bones in orthotopic xenograft mice using androgen receptor negative PC-3 cells. Ras GTPase-activating-like protein IQGAP1 was identified and validated as a hypercholesterolemia-induced metastasis-associated protein. Moreover, we show that knockdown of IQGAP1 or caveolin-1 in PC-3 cells abolished the hypercholesterolemia-induced metastasis from xenografts, indicative of their functional involvement in prostate cancer metastasis *in vivo*. IQGAP1 is a membrane-cytoskeleton scaffolding protein implicated in the progression of several types of tumors, potentially by regulating the Ras-MAP kinase and rac-cdc42 pathways [14-16]. Recruitment of IQGAP1 to plasma membrane ruffles facilitates cell migration [15], however, a role for cholesterol-enriched membranes in IQGAP1 regulation has not been examined.

## RESULTS

### Diet-induced hypercholesterolemia accelerates xenografted tumor metastasis

**Figure 1:**
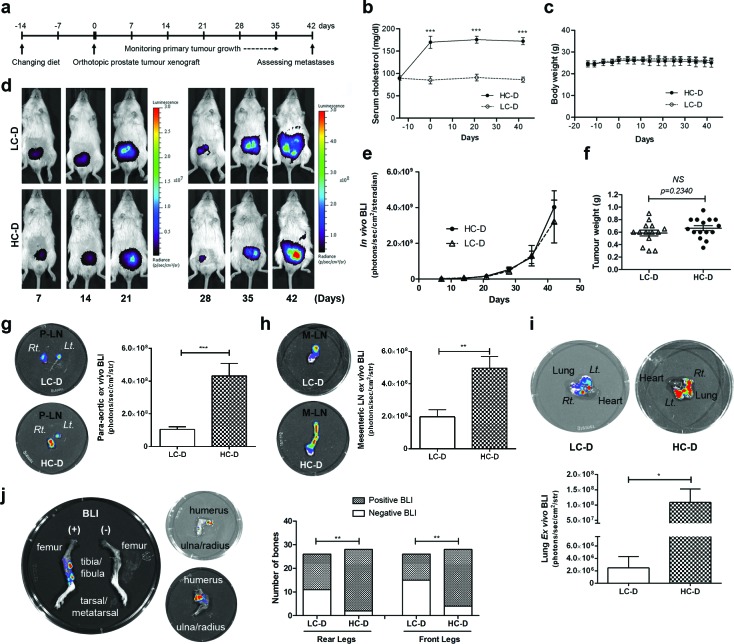
Diet-induced hypercholesterolemia promotes androgen-independent prostate cancer metastasis but not primary tumor growth (a) Experimental plan. Fourteen days prior to xenografting, male NOD/SCID mice were randomly allocated to low-cholesterol normal diet (LC-D, *n* = 14) or to a hypercholesterolemic diet (HC-D, *n* = 15). On day 0, human prostate cancer PC-3 expressing the luciferase gene were orthotopically injected into the dorsolateral prostate glands. (b) Serum cholesterol, (c) body weight and (d and e) primary tumor growth was measured by *in vivo* bioluminescence imaging on the indicated days during the course of the experiment. (f) At the end of the experiment, primary prostate tumors were weighed after removing seminal vesicles and the urinary bladder. *Ex vivo* imaging was used to measure metastasis in (g) para-aortic lymph node (P-LN), (h) mesenteric lymph node (M-LN), (i) lung, and (j) bone. Error bars show standard error of the mean. NS, not significant; *p < 0.05; **p < 0.005; ***p < 0.0005. Group comparison in (j) used Fisher's exact test, **p < 0.005.

To determine the direct but androgen-independent effects of hypercholesterolemia on advanced prostate cancer progression, we used an androgen-receptor negative prostate cancer cell line PC-3, expressing the luciferase gene (Supplementary Figure S1) in an orthotopic xenograft mouse model. Male NOD/SCID mice were randomly assigned to low-cholesterol normal (LC-D) and isocaloric hypercholesterolemic diet (HC-D) groups. Two weeks after diet initiation, *in vivo* and *ex vivo* studies were performed as illustrated in (Figure 1a). Similar to previous studies [7, 10], serum cholesterol levels (Figure 1b) but not body weights (Figure 1c) were significantly increased in the HC-D group. However, the intensity of *in vivo* bioluminescence (*p* = 0.87, Figure 1d and 1e) and final prostate tumor weights (*p* = 0.23, Figure 1f) were not significantly different between the groups, although the HC-D group showed an increased trend.

*Ex vivo* bioluminescence imaging was used to assess metastasis from the xenograft tumors [17]. Previous studies have reported PC-3 metastases including microscopic lung and bone metastases [18, 19]. The HC-D group showed higher bioluminescence intensity in the para-aortic lymph node (*p* = 0.0007, Figure 1g), mesenteric lymph node (*p* = 0.0021, Figure 1h) and the lung (*p* = 0.0275, Figure 1i). Bone metastasis is the major cause of prostate cancer related death [20] and previous studies have reported microscopic bone metastases from PC-3 xenograft tumors [18]. Indeed, 42% of bones collected from the LC-D group showed *ex vivo* positive bioluminescence (Figure 1j). Consistent with lymph node and lung metastases, an increased number of bones demonstrated positive bioluminescence in mice in the HC-D group in both the front (p = 0.0036) and rear legs (p = 0.0014, Figure 1j). Lymph node metastases and microscopic metastases of lung and bone marrow as well as metastasis in joint tissues were confirmed by histopathology (Supplementary Figure S2). Taken together, these results demonstrate that diet-induced hypercholesterolemia increased PC-3 tumor metastasis but not primary tumor growth.

### Diet-induced hypercholesterolemia is associated with increased IQGAP1 and caveolin-1 in the tumor

**Figure 2:**
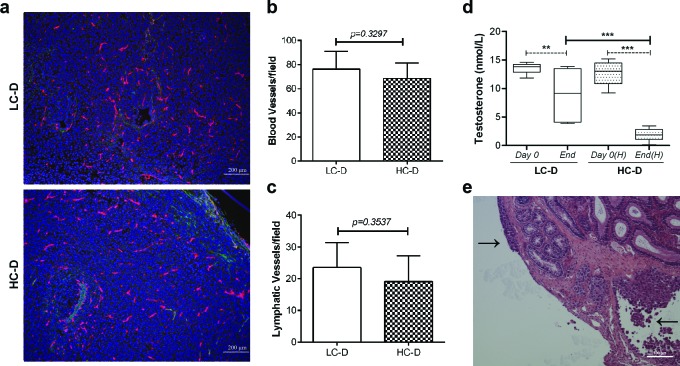
Diet-induced hypercholesterolemia is not associated with increased angiogenesis, lymphangiogenesis, and circulating testosterone levels (a) Representative images show double immunofluorescence staining of tumor tissue sections using the panendothelial cell marker endomucin (red) and the lymphatic specific marker podoplanin (green). Bar, 200 μm. (b) Angiogenesis was not significantly different between hypercholesterolemic and control diet groups (*p* = 0.3297, *n* = 9 per group). (c) Immunostaining for lymphangiogenesis demonstrated no significant difference between the diet groups (*p* = 0.35, *n* = 9 per group). (d) Circulating testosterone level was measured at days 0 and 42 using a homogeneous time resolved fluorescence assay. Data were represented as mean ± SEM (**p < 0.005, ***p < 0.0005). (e) Histopathology using H&E tissue sections demonstrated tumor cells invading into the capsule of the testes (arrows, tumors). Bar, 100 μm.

Metastatic prostate cancer is one of the most common causes of cancer-related death in men [21]. To identify the molecular mechanism which mediates hypercholesterolemia-stimulated prostate cancer metastasis, we first evaluated angiogenesis and hormone receptor signaling, based on recent reports [3-6, 8, 22]. Tumor angiogenesis and lymphangiogenesis were examined by staining primary tumor sections with the panendothelial cell marker endomucin and the lymphatic specific marker podoplanin (Figure 2a). No significant difference in angiogenesis (Figure 2a and 2b) or lymphangiogenesis (Figure 2a and 2c) was observed. While PC-3 cells lack androgen receptor expression, it was possible that hypercholesterolemia increased circulating testosterone levels which could positively affect tumor stroma tissues. Hence we measured serum testosterone levels (Figure 2d). Surprisingly, serum testosterone levels were significantly decreased in both diet groups at the conclusion of the experiment (*p* = 0.016 in LC-D and *p* < 0.0001 in HC-D, Figure 2d) which might be due to aggressive invading tumor cells from the primary and secondary tumors to the testis and surrounding organs/tissues (Figure 2e and Supplementary Figure S3). Additionally, hypercholesterolemia was associated with a greater reduction in circulating testosterone levels, possibly due to negative feedback in impaired testicular functions [23, 24].

**Figure 3:**
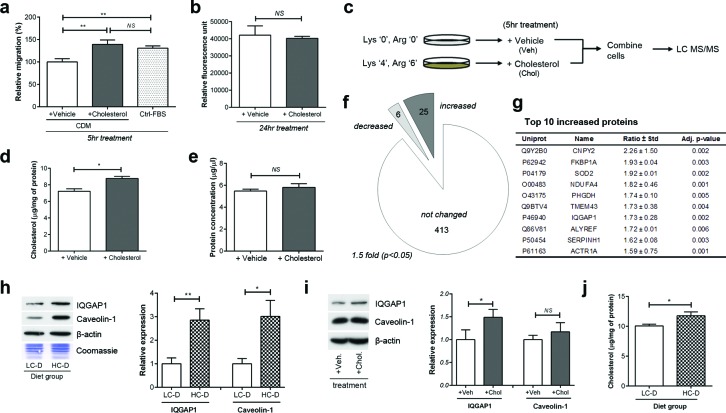
*In vitro* quantitative proteomics suggests IQGAP1 as a cholesterol-related candidate (a) PC-3 transmigration assay was performed using cholesterol-deficient medium (CDM) or control FBS (Ctrl-FBS). (b) PC-3 proliferation was determined using Alamar Blue assay. (c) Experimental plan for *in vitro* quantitative proteomics study using stable isotope labeling by amino acids in cell culture (SILAC). (d) Total cellular cholesterol and (e) protein concentrations of whole cell lysates were measured. (f) Cholesterol treatment (5 μM for 5 hours) significantly altered 41 out of 441 proteins, based on a 1.5 fold difference and *p*-values less than 0.05. (g) Top 10 protein candidates up-regulated by cholesterol treatment. Uniprot, UniProt Entry ID; Name, gene name; Ratio ± STD, cholesterol(heavy)/control(light) SILAC ratio ± standard deviation; Adj. p-value, permutation p-value adjusted for multiple testing. (h) Immunoblotting of tumor lysates show that expression levels of IQGAP1 and caveolin-1 were significantly increased in HC-D group (*n* = 9 per group). (i) PC-3 cells were incubated with 5 μM cholesterol or vehicle for 5 hours. Total cell lysates (20 μg) were separated on SDS-PAGE and immunoblotted with antibodies against IQGAP1, caveolin-1 and β-actin as indicated. IQGAP1 but not caveolin-1 was significantly altered by short-term cholesterol treatment. (j) Intratumoral cholesterol levels was measured in tumor lysates using Amplex Red cholesterol assay (*n* = 9 per group). Error bars show standard error of the mean; NS, not significant; *p < 0.05; **p < 0.005.

To identify metastasis-promoting proteins induced by cholesterol, we performed *in vitro* quantitative proteomics using stable isotope labeling by amino acids in cell culture (SILAC). Initial experiments showed that long-term growth of cell culture in reduced-cholesterol media affected cell proliferation, anchorage-independent growth, as well as migration (Supplementary Figure S4). To mimic the *in vivo* results of increased metastasis but not primary tumor growth, we selected a short-term, 5 hour treatment which increased PC-3 transmigration (Figure 3a) but not proliferation (Figure 3b). Therefore, SILAC was performed according to the scheme in (Figure 3c).

Compared to cells maintained in cholesterol-deficient medium (CDM) containing 5% delipidated fetal bovine serum (FBS), incubation with additional 5 μM cholesterol for 5 hours significantly altered total cellular cholesterol level (Figure 3d) but not total cellular protein amount (Figure 3e). Liquid chromatography-tandem mass spectrometry and database searching identified and quantified a total of 441 proteins from 3 biological replicates. Significance of SILAC ratios was determined by permutation test [25-27]. By applying a 1.5 fold change filter for biological significance, 31 proteins were deemed to be significantly altered by cholesterol supplementation (Figure 3f and, Supplementary Table S1 and S2). The top ten proteins up-regulated by short-term cholesterol treatment included Ras GTPase-activating-like protein IQGAP1 (Figure 3g), a protein implicated in tumor metastasis in several cancers [16]. IQGAP1 is a scaffolding protein which interacts with Rac1 and Cdc42, promoting actin polymerization at the leading edge of migrating cells [28]. As there are no reports linking hypercholesterolemia or prostate cancer with IQGAP1, we went on to confirm this result *in vitro* and *in vivo*. In parallel with IQGAP1, we also measured the level of caveolin-1 as it is associated with metastasis and poor prognosis in prostate cancer [19, 29] and has been functionally linked with IQGAP1[30-32].

We performed Western blotting of both xenograft tumor and cell lysates to determine the effect of hypercholesterolemia on the expression of level of caveolin-1 and IQGAP1. Interestingly, xenograft tumor lysates from the HC-D group showed higher expression of both IQGAP1 (2.85 ± 0.50, *p* = 0.003) and caveolin-1 (3.0 ± 0.7, *p* = 0.011, Figure 3h), although cholesterol treatment of PC-3 cells *in vitro* increased expression of IQGAP1 but not caveolin-1 (Figure 3i). Cholesterol elevation in the tumor was confirmed (Figure 3j). The differences between *in vivo* and *in vitro* data may relate to the different duration of hypercholesterolemia, presence of stromal cells, tissue architecture and/or feedback mechanisms *in vivo*.

### Cholesterol and caveolin-1 recruit IQGAP1 to lipid organized membrane microdomains

**Figure 4:**
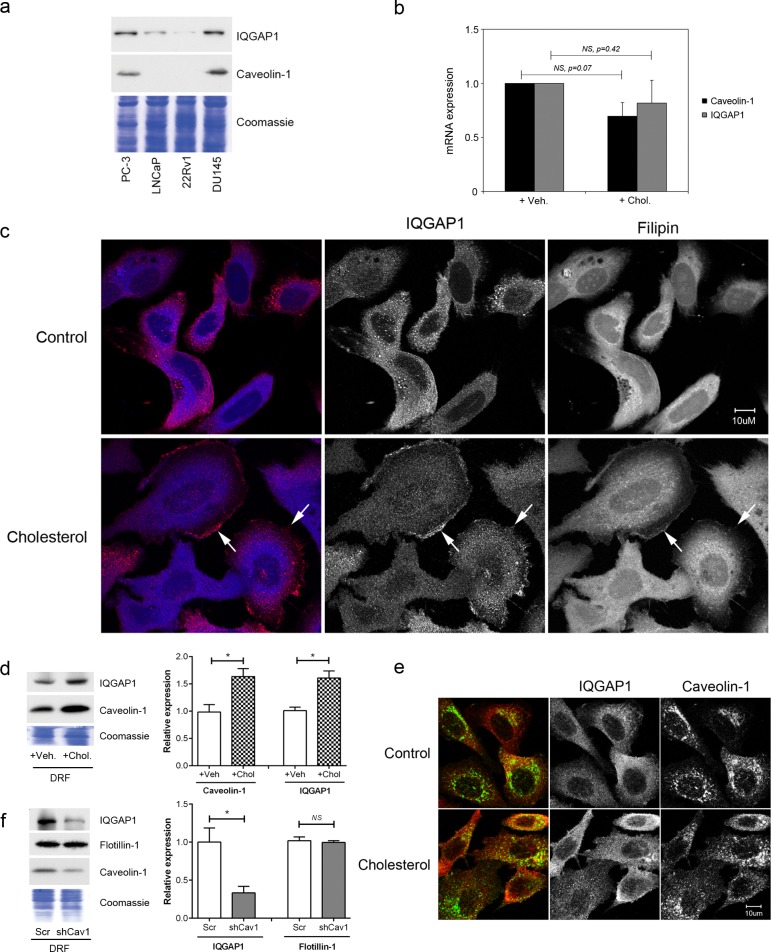
Cholesterol regulates IQGAP1 targeting to detergent-resistant fractions (a) Relative expression of IQGAP1 and caveolin-1 in prostate cancer cell lines. Total cell lysates (20 μg) from PC-3, LNCaP, 22Rv1 and DU145 were resolved on SDS-PAGE and immunoblotted for IQGAP1 and caveolin-1. Loading control: Coomassie staining. (b) Cholesterol treatment for 5 hours does not alter caveolin-1 or IQGAP1 transcription as measured by qRT-PCR. (c) Immunofluorescence labeling for IQGAP1 (red) and staining for cholesterol using Filipin III (blue) in PC-3 cells, after treatment with 5 μM cholesterol or vehicle for 20 hours. Arrows, membrane ruffles enriched in cholesterol and IQGAP1. Bars, 10 μm. (d) IQGAP1 levels in detergent-resistant fractions (DRF) was examined in PC-3 cells (*n* = 3). (e) Immunofluorescence co-labeling for caveolin-1 and IQGAP1 in PC-3 cells after 20 hour treatment with 5 μM cholesterol or vehicle. Bars, 10 μm. (f) DRF was collected from equal numbers of caveolin-1 knockdown (shCav1) and control scrambled sequence knockdown (Scr) PC-3 cells. Equivalent volumes of DRF were separated by SDS-PAGE and immunoblotted for IQGAP1, flotillin-1 or caveolin-1. *n* = 3. Error bars show standard error of the mean; NS, not significant; *p < 0.05. Veh, vehicle; Chol, cholesterol.

Having established IQGAP1 as a cholesterol-induced protein in PC-3 cells, we next examined its expression in other commonly-used prostate cancer cell lines (Figure 4a). In agreement with a role in mediating metastasis, the level of IQGAP1 is higher in aggressive prostate cancer cells, PC-3 and DU145, which also express caveolin-1 (Figure 4a). Compared to PC-3 cells (Figure 3i), caveolin-1-negative LNCaP cells showed a delay in time-response of cholesterol-induced IQGAP1 elevation, which was significant at 20 hours but not at 5 hours post cholesterol addition (Supplementary Figure S5). These results suggest an association between cholesterol-sensitivity and aggressiveness of prostate cancer cell lines, with IQGAP1 being a potential downstream effector.

Cholesterol regulates gene expression *via* two transcription factors, sterol regulatory element binding protein (SREBP) and liver X receptor (LXR). Hence, we tested the hypothesis that hypercholesterolemia increases IQGAP1 protein at the transcriptional level using qPCR. Surprisingly, IQGAP1 mRNA was not responsive to cholesterol or oxysterol treatment in PC-3 and LNCaP cells (Figure 4b and Supplementary Figure S6), indicating that hypercholesterolemia increases IQGAP1 protein at a post-transcriptional level.

Next, we investigated if hypercholesterolemia altered membrane localization of IQGAP1, which mediates cell migration [15]. Dynamic membrane re-organization occurs during cell migration and is regulated by caveolin-1 via cholesterol-rich membranes (also called lipid rafts), partly through its role as the structural protein of plasma membrane pits caveolae [1, 30]. While IQGAP1 is known to be enriched in membrane ruffles, association with cholesterol-rich membranes has not been examined. We evaluated the effect of cholesterol treatment on the localization of IQGAP1 in PC-3 cells (Figure 4c). IQGAP1 showed diffuse and punctate staining in control cells. After 20 hour treatment in cholesterol, we observed increasing localization of IQGAP1 to membrane ruffles (Figure 4c, arrows) which also stained with filipin, an antibiotic which stains cholesterol. These results suggest that cholesterol induced the translocation of IQGAP1 to cholesterol-rich membranes in the ruffles. To further investigate this, we employed a biochemical method of preparing cholesterol-rich membranes, previously established as detergent resistant fractions (DRFs) [33]. Caveolin-1 is a known membrane cholesterol-binding protein and served as a positive control for the fractionation. IQGAP1 was detected in PC-3 DRF (Figure 4d). Both caveolin-1 and IQGAP1 levels in DRF were increased by cholesterol treatment (Figure 4d).

Previous studies suggest a functional interaction between caveolin-1 and IQGAP1 in regulating the ERK [31] and integrin-linked kinase signaling pathways [32]. Our results show that hypercholesterolemia increases the level of both proteins in the DRF fraction, but it is not clear whether caveolin-1 and IQGAP1 actually co-localize to the same membranes. To address this question, we performed immunofluorescence co-localization with and without cholesterol treatment (Figure 4e). Some co-localization was observed, but the effect of a 20 hour cholesterol treatment was minor (mean Pearson's R-values of 0.35 and 0.36 for control and cholesterol, respectively). In view of the previous publications on the functional interaction between caveolin-1 and IQGAP1 [31, 32], we also investigated if caveolin-1 is required for recruitment of IQGAP1 to DRF (Figure 4f). Interestingly, we observed a reduced level of DRF IQGAP1 in shCav1 PC-3 cells, while another cholesterol-rich membrane microdomain-associated protein, flotillin-1, was not affected (Figure 4f). These results suggest that caveolin-1 indirectly regulates IQGAP1-membrane association, potentially by modulating membrane lipid organization [34].

### IQGAP1 and caveolin-1 facilitate PC-3 metastasis

**Figure 5:**
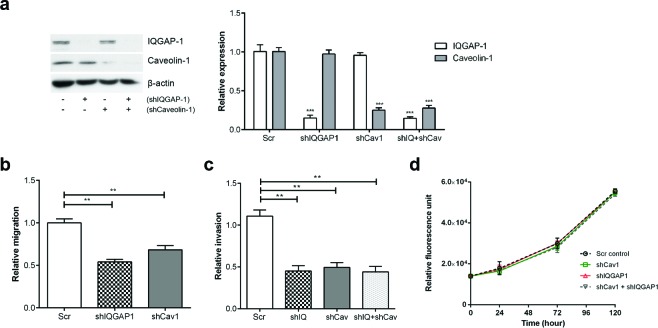
Knockdown of IQGAP1 and/or caveolin-1 in PC-3 cells reduces migration and invasion *in vitro* PC-3-luciferase cell lines with reduced expression of IQGAP1 (shIQGAP1), caveolin-1 (shCav1) and both (shIQ+shCav) were generated using lentiviral-mediated shRNA. (a) Relative protein expression of IQGAP1 and caveolin-1 was measured by immunoblotting and quantified over 3 experiments. (b) Transmigration, (c) Matrigel invasion and (d) proliferation assays were performed for PC-3 stable cell lines. Error bars show standard error of the mean. **p < 0.005, ***p < 0.0005. Additional shRNAs produced similar phenotypes (Supplementary Figure S7).

To further evaluate the involvement of IQGAP1 and caveolin-1 in PC-3 metastases *in vivo*, we performed hypercholesterolemic diet xenograft experiments using PC-3-luc cell lines with reduced IQGAP1 and/or caveolin-1 expression. Firstly, pooled populations of shIQGAP1 and shCav1 PC-3 cells were established using lentiviral mediated shRNAs and flow cytometry (based on the bicistronic co-expression of GFP in the lentivirus). A double knockdown cell line was generated by infecting shIQGAP1 cells with shCav1 lentivirus. Reduced protein levels were confirmed by Western blotting (Figure 5a). Preliminary *in vitro* studies showed similar attenuation of transmigration and invasion between shIQGAP1, shCav1 and double knockdown cells compared to scrambled control (Figure 5b, 5c and Supplementary Figure S7). There was no difference in proliferation between the 4 cell lines (Figure 5d). Before performing *in vivo* study, the intensity and linearity of *in vitro* bioluminescence of PC-3-luc shIQGAP1 and shCav1 cell lines were confirmed for accurate comparison between groups (Supplementary Figure S8).

**Figure 6:**
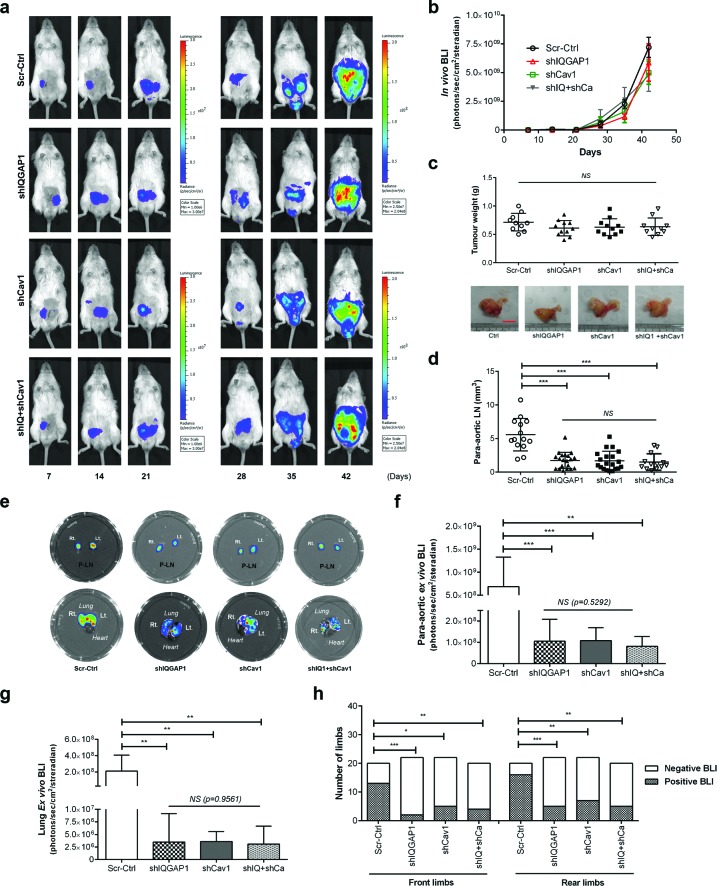
Knockdown of IQGAP1 and/or caveolin-1 suppressed hypercholesterolemia-induced prostate cancer metastasis *In vivo* metastasis was assayed for shIQGAP1, shCav1 and shIQ+shCav1 PC-3-luciferase cells in HC-D intraprostatic xenograft model. (a and b) Real-time xenografted primary prostate tumor growth was monitored using whole body *in vivo* bioluminescence. Scrambled control (Scr-Ctrl, *n* = 10), IQGAP1 knockdown (shIQGAP1, *n* = 11), caveolin-1 knockdown (shCav1, *n* = 11), and double knockdown of IQGAP1 and caveolin-1 (shIQ+shCav1, *n* = 10). (c) Final prostate tumor weight was assessed after removing seminal vesicles and urinary bladder. Representative figures are shown (Bar, 10 mm). (d) The size of para-aortic lymph nodes was measured. (e) Representative figures show *ex vivo* bioluminescence imaging for para-aortic lymph nodes (P-LN) and the lung tissues. *Ex vivo* bioluminescence was measured for secondary metastases in (f) para-aortic lymph nodes, (g) lung and (h) bone. Error bars show standard error of the mean. NS, not significant; *p < 0.05, **p < 0.005, ***p < 0.0005.

A total of 42 male NOD/SCID mice were randomly assigned to and xenografted with scrambled control (Scr-Ctrl), IQGAP1 knockdown (shIQGAP1), caveolin-1 knockdown (shCav1) and double knockdown (shIQ+shCa) PC-3 cells. *In vivo* and *ex vivo* studies were performed as described in Figure 1a using HC-D. As expected, all groups showed similar body weight (*p* = 0.21, Supplementary Figure S9a) and serum cholesterol levels (*p* = 0.96, Supplementary Figure S9b). Whole body *in vivo* imaging showed similar primary tumor growth curves (*p* = 0.55, Figure 6a and 6b), and final prostate tumor weight also revealed no significant changes (*p* = 0.39, Figure 6c) between the groups. Tumor metastases were determined in lymph nodes, lung and bones including joint tissues. Measurement of lymph node size revealed significantly smaller lymph nodes in knockdown groups compared to control (*p* < 0.0005), but not among knockdown groups (*p* = 0.8809, Figure 6d). Similarly, *ex vivo* imaging demonstrated significantly reduced lymph node metastases (Figure 6e and 6f) as well as microscopic lung metastases (Figure 6e and 6g). Pairwise comparisons using Fisher's exact test also demonstrated significantly reduced metastatic invasion to the bones and joints in shIQGAP1 (front limbs; *p* = 0.0003, rear limbs; *p* = 0.0005), shCav1 (*p* = 0.012 and *p* = 0.0023, respectively), or double knockdown PC-3 cells (*p* = 0.0095 and *p* = 0.0012, respectively, Figure 6h). Double knockdown of IQGAP1 and caveolin-1 showed no additive effects in the reduction of metastases (Figure 6f, 6g and 6h) suggesting action *via* the same molecular pathway. Taken together, these results suggest that IQGAP1 and caveolin-1 potentiate PC-3 metastasis, and that the elevation of IQGAP1 levels in hypercholesterolemia plays an important role in metastasis.

## DISCUSSION

Androgen-deprivation therapy is the first line of treatment for advance prostate cancer. While highly effective in androgen-sensitive disease, resistance ultimately develops in most patients [13]. Androgen-deprivation therapy is associated with a spectrum of side effects, including elevated circulating cholesterol [12] which may paradoxically promote androgen-independent prostate cancer metastasis based on this study using a xenograft mouse model. Our results indicate that in addition to the recently reported hormone receptor and cholesterol metabolism dependent mechanisms [3, 7] hypercholesterolemia *per se* can increase prostate cancer metastasis by modulating the properties of membrane microdomains. We further demonstrate the involvement of two proteins, caveolin-1 and IQGAP1 in hypercholesterolemia-induced xenograft tumor metastasis *in vivo*. Hence, caveolin-1, IQGAP1, or cholesterol-rich membrane microdomains (lipid rafts) are potential therapeutic targets for prevention or treatment of tumor metastasis.

Caveolin-1 is a cholesterol binding protein well characterized by its role in formation of plasma membrane pits, caveolae [30]. Caveolin-1 has been considered a biomarker and therapeutic target in prostate cancer as it is released from prostate cancer cells, can be detected in the serum and positively associates with prostate cancer grade [29, 35, 36]. However, caveolin-1 plays complex roles in cancer, with no existing models to explain its apparent contradictory tumor promoter and suppressor roles. Recent studies have begun to reveal the complexity of caveolin-1 function in cancer. Firstly, caveolin-1 may function pathologically outside caveolae *via* non-caveolar caveolin-1 membrane raft domain (caveolin-1 microdomains), as discovered in PC-3 cells [37] and validated in prostate cancer tissue samples [19]. While caveolin-1 microdomains (not caveolae) are aberrantly induced in advanced prostate cancer cells, caveolae is reduced in the stroma during prostate cancer progression [19]. Given the complexity of caveolin-1 cellular and subcellular expression during cancer progression, therapeutic targeting of caveolin-1 is challenging. Furthermore, the molecular mechanisms of caveolin-1 in cancer progression have not been fully elucidated, although it likely involves modulation of lipid rafts and, based on the present data, IQGAP1.

IQGAP1 is a ubiquitously expressed scaffolding protein that co-ordinates spatial signaling to regulate mitogenic, morphological and migratory processes [14]. Recent studies implicate a role for IQGAP1 in cancer, with overexpression reported in ovarian [38], liver [39], advanced colorectal [40], thyroid [41], pancreatic cancer [42] and squamous cell carcinoma [16]. Consistent with the known ubiquitous expression of IQGAP1, we detected it in all 4 commonly used prostate cancer cell lines (Figure 4a). Interestingly, IQGAP1 expression level correlated with aggressiveness of the cell lines, and shows correlation with caveolin-1, a known regulator of cholesterol membrane microdomains (Figure 4a). Furthermore, loss of caveolin-1 expression attenuated the level of IQGAP1 in the detergent resistant fraction (Figure 4f), indicative of caveolin-1-dependent recruitment to cholesterol-rich membrane domains. While IQGAP1 membrane targeting is essential for its regulation of migration, to our knowledge, this is the first report of IQGAP1 targeting to cholesterol-rich membrane microdomains. Since cholesterol is essential for all cell types, and IQGAP1 is ubiquitously expressed, we asked if IQGAP1 targeting to cholesterol-rich lipid rafts can be detected in other cancer cells. By scrutinizing 66 mass spectrometry-based human lipid raft proteomics studies published between 2001 and 2013 using RaftProt database [43], we found that IQGAP1 was identified in 12 experiments which included 5 cancer cell lines, namely HeLa cervical cancer, SW620 and SW480 colorectal cancer, NG108-15 neuroblastoma-glioma hybrid, and Jurkat acute T cell leukemia [44-48]. Both detergent-based and detergent-free lipid raft extraction methods were featured in these experiments, indicating that the presence of IQGAP1 was not an isolation artifact. Furthermore, sensitivity to the cholesterol-depleting agent methyl-β-cyclodextrin suggests that IQGAP1 is a *bona fide* lipid raft protein.[48] Interestingly, lipid raft IQGAP1 level was elevated in a metastatic colorectal cancer cell line, compared to a non-metastatic cell line derived from the same patient [45]. On the other hand, CCL20 and cisplatin treatment did not affect lipid raft IQGAP1 levels in Jurkat cells [46, 47]. Taken together, these data add support to our experimental results showing that IQGAP1 targeting to lipid raft membranes can be regulated and may contribute to cancer metastasis.

In addition to IQGAP1, it is likely that other cholesterol-rich membrane microdomain-associated proteins are altered by hypercholesterolemia to impact on tumor metastasis. Some examples include CD44, ezrin, uPAR and MMP-9 [49, 50]. This study adds alteration of cholesterol-rich microdomain composition and function to the increasing battery of molecular mechanisms linking hypercholesterolemia and cancer development and progression. Our results also highlight the importance of monitoring and maintaining low to normal blood cholesterol levels for prostate cancer patients, particularly those undergoing androgen deprivation therapy which can elevate cholesterol [12].

## MATERIALS AND METHODS

### Cell lines

Human prostate cancer cell lines PC-3 and LNCaP were purchased from American Type Culture Collection (ATCC). Human prostate cancer cell lines DU145, and PC-3 cells stably expressing firefly luciferase genes[51] were kindly provided by Dr. Ming-Tat Ling (Australian Prostate Cancer Research Centre–Queensland and Institute for Biomedical Health & Innovation, Queensland University of Technology, Translational Research Institute, Brisbane, Australia). The prostate cancer cell line 22Rv1 was kindly provided by Dr. John Hooper (Mater Medical Research Institute, Translational Research Institute, Brisbane, Australia). Prostate cancer cell lines were grown in RPMI-1640 medium (Invitrogen) supplemented with 5% fetal bovine serum (FBS).

### Reagents

Xenolight D-luciferin potassium salt was obtained from ThermoFisher. Standard (low-cholesterol) control diet and hypercholesterolemic diet (SF12-007, equivalent to TD88051, Harlan[10]) were purchased from Specialty Feeds (Glen Forrest, WA, Australia). Cholesterol levels were measured using Amplex Red cholesterol Assay Kit (Invitrogen), and fluorescence was measured by FLUOstar OPTIMA (BMG Labtech). Serum testosterone level was measured using homogeneous time resolved fluorescence based total testosterone assay kit (Cisbio) with a microplate reader, Artemis 101 (Cosmo Bio). Anti-caveolin-1 (610407) and anti-flotillin-1 (610821) antibodies were from BD Transduction Laboratories, anti-IQGAP1 (22167-1-AP) from ProteinTech, anti-β-actin (A1978) from Sigma, anti-endomucin (sc-53941) from Santa Cruz Biotechnology and anti-podoplanin (RDI-103M40X) from Bioclone Aust. Alexa-fluor secondary antibodies were from Invitrogen.

### Orthotopic prostate cancer xenograft and monitoring mice

Use of mice and all animal experiments described in this paper were conducted after prior approval by the University of Queensland Animal Ethics Committee (UQDI/326/10/AICR). Seven-week-old male NOD/SCID mice were purchased from the Animal Resource Centre (Canning Vale, WA, Australia), housed and maintained at the University of Queensland Biological Resource Animal Facility.

All animal experiments including *in vivo* and *ex vivo* imaging were performed by two veterinarians as previously described [19]. Prior to xenograft experiments, the *in vitro* bioluminescence (BLI) sensitivity and linearity of PC-3-luciferase cell lines were confirmed (Supplementary Figures S1 and S8). Briefly, 2.5 × 10^5^ PC-3-luciferase cells were orthotopically injected into the dorsolateral prostate glands of male NOD/SCID mice under a dissecting microscope. Prior to *in vivo* bioluminescence imaging, D-luciferin dissolved in PBS was intraperitoneally injected, and then mice were anesthetized under isoflurane. At the end of the final experiments, mice were euthanized, and organs were collected in small 6 to 10 cm dishes containing PBS for *ex vivo* imaging. All *ex vivo* imaging was finished within 15 minutes. The size of lymph nodes was measured using a caliper [tumor volume = (width)^2^ × length/2]. Mouse tissues were fixed in 10% neutral-buffered formalin for 24 hours, and bone tissues were decalcified in 10% EDTA for more than 2 weeks. Histopathological changes in mouse tissues were carefully examined by a veterinary pathologist. In order to examine serum cholesterol and testosterone levels, retro-orbital sinus bleeding and cardiac puncture were used after diet restriction for 2 hours.

### Angiogenesis and lymphangiogenesis

Paraffin-embedded sections were used for staining with the panendothelial cell marker endomucin and the lymphatic specific marker podoplanin. The staining and quantification of the lymphatic and blood vessel densities for 5 randomly selected fields for each slide was performed as described previously (*n* = 9 per each) [52]. Nuclei were stained using 4′,6-Diamidino-2-phenylindole dihydrochloride (DAPI). Slides were mounted on coverslips with 70% glycerol in PBS solution.

### Cholesterol-deficient medium and cholesterol loading *in vitro*

To modulate cellular cholesterol levels *in vitro*, delipidated FBS and dialyzed FBS (control FBS) were used. Delipidated FBS was prepared by extracting lipids from FBS using diisopropylether and n-butanol in a ratio of 2:1. After centrifugation at 350 g for 15 minutes at 4°C, the supernatant was removed. FBS re-dissolved in diisopropylether in 1:1 ratio was centrifuged again at 250 g for 15 minutes at 4°C. Lastly, the supernatant was dialyzed against cold PBS using a 3500 MWCO dialysis membrane. Medium containing 5% delipidated FBS was used as cholesterol-deficient medium, and medium containing 5% dialyzed FBS without delipidation was used as control medium. Cholesterol (Sigma-Aldrich) was dissolved in ethanol, and 5 μM cholesterol or ethanol (vehicle) were added into cholesterol-deficient medium.

### *In vitro* quantitative proteomics using stable isotope labeling by amino acids in culture (SILAC)

SILAC labeling of PC-3 cells was performed as previously described [25], with the following amino acids: “0/0” cells with normal isotopic Lys and Arg and “4/6” cells with ^2^H_4_-Lys and ^13^C_6_-Arg. Greater than 99% incorporation was confirmed for the “4/6”-labelled cells by liquid chromatography tandem mass spectrometry (LC-MS/MS).

After cholesterol treatment, cells were washed twice in PBS and scraped in PBS. Cell pellets were obtained by centrifugation at 220 g, then lysed in a modified RIPA buffer: 20 mM Tris pH 7.5, 150 mM NaCl, 0.5% Triton X-100, 0.5% sodium deoxycholate, 10 mM sodium fluoride, 0.5 mM sodium vanadate, 1 mM sodium pyrophosphate, 0.5 mM 4-(2-aminoethyl) benzenesulfonyl fluoride hydrochloride and protease inhibitors containing 1 μg/μL Aprotinin, 1 μg/μL Antipain, 1 μg/μL Pepstatin, 1 μg/μL Leupeptin and 2.5 mM Benzamidine. Insoluble material was removed by pelleting at 12,000 g for 5 minutes at 4°C. The supernatant was collected as total cell lysate and the protein concentration was determined using Bradford assay (Biorad).

### In gel digest and tandem mass spectrometry

Semi-automated in-gel tryptic digestion was performed on a liquid handler as previously described [53]. Peptides were analyzed using a 1200 Series nano high performance liquid chromatography coupled with Quadrupole-Time of Flight 6520 with a Chip-Cube interface (Agilent Technologies) as described [53]. Spectrum Mill (Agilent, B.04.00.127) was used for data analysis. Data were extracted with carbamidomethylated cysteine as a fixed modification and SILAC amino acids N-Lys, ^13^C_6_^15^N_2_-Lys, N-Arg and ^13^C_6_^15^N_4_-Arg as a mix modification. Extracted data were searched against the SwissProt (release-2011_01) human database with the same modifications as above, and oxidized methionine as a variable modification. Precursor and product mass tolerance was set to ± 20 ppm and ± 50 ppm respectively. Reverse database scores were calculated with Spectrum Mill search engine by using the percentage of false positive identifications using the following cut-off: protein score > 11, peptide score >10 and scored peak intensity > 60%. All peptides identified had a global false discovery rate of less than 0.9%. Mean SILAC ratio (H/L) and standard deviations were calculated using all the peptide ratios matched to a protein, and p-values were calculated using peptide SILAC ratios as previously described [25-27].

### Immunofluorescence

Cells were grown on coverslips and fixed in 4% paraformaldehyde for 30min followed by three washes in PBS. Where stated, cells were stained with Filipin III (Sigma) at a concentration of 50μg/ml in PBS for 2 hours followed by three washes in PBS. Cells were then permeabilized with 0.1% saponin and 5% BSA in PBS for 30min. Cells not stained with filipin were permeabilized in 0.1% Triton-X100 and 5% BSA in PBS for 30min, followed by 3 washes in PBS. The coverslips were incubated with the primary followed by secondary antibodies diluted in 3% BSA/PBS for 1hr each at room temperature. Coverslips were washed 3 times in PBS after the addition of each antibody. Where stated, coverslips were counterstained with 1μg/mL DAPI, washed three times with PBS, rinsed in MilliQ water and mounted in ProLong Diamond (Invitrogen). Cells were imaged by confocal microscopy (Ziess 510 Meta or Olympus FV1200). Pearson's R value was calculated for 50 individual cells in ImageJ using the Coloc2 plugin.

### Generation of knockdown cell lines

In order to knockdown caveolin-1 and/or IQGAP1 expression, lentiviral particles expressing shRNAs targeting IQGAP1 or caveolin-1 were obtained from the University of Queensland Institute for Molecular Bioscience, Life Science Automation Facility. The sequence of shRNAs for IQGAP1 was: shIQGAP1 (sIQ1), TGCTGTTGACAGTGAGCGATCCCACAAAGATGAAGTTGTAT AGTGAAGCCACAGATGTATACAACTTCATCTTTGTGGGAGTGCCTACTGCCTCGGA; shIQGAP1 (sIQ2), TGCTGTTGACAGTGAGCGCAAGGTTGACTTCACAGAAGAATAGTGAAGCCACAGATGTATTCTTCTGTGAAGTCAACCTTTTG CCTACTGCCTCGGA; shIQGAP1 (sIQ3) TGCTGTTGACAGTGAGCGATCGAAGGTAGATCAG ATTCAATAGTGAAGCCACAGATGTATTGAATCTGATCTACCTTCG ACTGCCTACTGCCTCGGA. The sequence of shCaveolin-1 was: shCaveolin-1 (sC1), TGCTGTTGACAGTGAGCGAAACGATGACGTGGTCAAGATTTAGTGAAGCCACAGATGTAA ATCTTGACCACGTCATCGTTGTGCCTACTGCCTCGGA; shCaveolin-1 (sC2), TGCTGTTGACAGTGAGCGCCACCACCTTCACTGTGACGAATAGTGAAGCCACAGATGTATTCGTCACAGTGAAGGTGGTGATGCCT ACTGCCTCGGA; shCaveolin-1 (sC3), TGCTGTTGACAGTGAGCGACACCTTCACTGTGACGAAATATAGTGAAGCCACAGATGTA TATTTCGTCA CAGTGAAGGTGGTGCCTACTGCCTCGGA. In preliminary studies, these different shRNAs were tested (Supplementary Figure 7a) with variable results. At least 2 of each shRNA construct (sIQ1, sIQ3; sC1, sC2) produced similar functional outcomes as measured by proliferation and transmigration assay (Supplementary Figure 7b, 7c). Thereafter, sIQ3 and sC2 were selected for producing double knockdown cells and for *in vivo* experiments (Figure 6, and shown in Figure 5 as representative).

### *In vitro* assays

All *in vitro* studies were repeated at least three times. PC-3 proliferation was determined using Alamar Blue (Invitrogen). At appropriate time points after plating 10,000 cells in 96-well plates, cells were incubated in non-phenol red RPMI medium containing 10% Alamar Blue for 3 hours at 37^o^C. The fluorescence was measured using a microplate reader, FLUOstar OPTIMA (BMG LabTech).

Transmigration assays were performed using 8 μm pore size transwell inserts (Corning) coated with collagen type I (Sigma-Aldrich) as previously described [54] with minor modification. Briefly 100 μl of cell suspension (2.5 × 10^6^ cells per mL) in serum-free RPMI media containing 0.1% BSA was added into the upper chamber. RPMI media containing 5% FBS was added to the lower chamber and the PC-3 cells were allowed to migrate for 5 hours. The filter was rinsed in PBS and fixed in 4% paraformaldehyde. Cells that did not migrate were removed by swabbing the membrane from the top side of the insert. Cells remaining on the membrane were stained with haematoxylin. Matrigel invasion assay was performed similarly. Matrigel (BD Biosciences) was mixed 1:1 with medium, and the mixture was added into the transwell inserts. Then, 100 μl of cell suspension (5 × 10^6^ cells per mL) in serum-free RPMI media containing 0.1% BSA was placed into the upper chamber, and then incubated at 37^o^C for 20 hours. The number of migrated or invaded cells was counted in 5 different fields using a bright field microscope.

### Subcellular fractionation

A detergent-resistant lipid-rich membrane fraction (DRF) was prepared as previously described [33], with minor modification. Briefly, PC-3 (1.5 × 10^6^) cells were plated in 10 cm dishes the day before harvest. Cell pellets were prepared by centrifugation at 220 g, and then homogenized in 1% Triton-X-100, 25mM Tris HCl, pH 7.4, 150mM NaCl, 3mM EDTA and protease inhibitors by passage through a 27-guage needle 25 times. The homogenate was incubated on ice for 30 minutes, and then centrifuged at 16,000 g for 20 minutes at 4^o^C. The insoluble pellets (detergent-resistant fraction) were resuspended in 40 μl SDS-PAGE sample buffer, and then 10 μl samples were loaded on SDS-PAGE gels for Western blots. Western blots were performed as previously described [19].

### Real-time RT-PCR

Cells were grown in triplicate on 12-well plates and treated as described in figure legends. Total RNA was harvested using TRI Reagent (Sigma-Aldrich), and reverse transcribed into cDNA using the SuperScript III First Strand cDNA Synthesis Kit (Life Technologies). mRNA levels were determined by qRT-PCR using SensiMix SYBR Green (Bioline) on a Corbett Rotorgene 3000 (Corbett Life Sciences, Sydney, Australia) using primers for *IQGAP1* (F: AAGAAGGCATATCAAGATCGG, R: CCTCAGCATTGATGAGAGTC), *ABCA1* [55], *HMGCR* [56] and the housekeeping gene, *porphobilinogen deaminase*, *PBGD* [55]. The mRNA expression levels were normalized to that of PBGD and made relative to the vehicle condition using the ΔΔCt method.

### Statistical analysis

Two-tailed statistical analysis was performed using the GraphPad Prism software, and p-value less than 0.05 was regarded as significant. Pair-wise comparison for positive or negative bioluminescence was performed using Fisher's exact test.

## SUPPLEMENTARY MATERIAL FIGURES AND TABLES







## References

[1] Moon H, Hill MM, Roberts MJ, Gardiner RA, Brown AJ (2014). Statins: protectors or pretenders in prostate cancer?. Trends in endocrinology and metabolism: TEM.

[2] Nielsen SF, Nordestgaard BG, Bojesen SE (2012). Statin use and reduced cancer-related mortality. The New England journal of medicine.

[3] Li X, Chen YT, Hu P, Huang WC (2014). Fatostatin Displays High Antitumor Activity in Prostate Cancer by Blocking SREBP-Regulated Metabolic Pathways and Androgen Receptor Signaling. Molecular cancer therapeutics.

[4] Nelson ER, Wardell SE, Jasper JS, Park S, Suchindran S, Howe MK, Carver NJ, Pillai RV, Sullivan PM, Sondhi V, Umetani M, Geradts J, McDonnell DP (2013). 27-Hydroxycholesterol links hypercholesterolemia and breast cancer pathophysiology. Science.

[5] Wu Q, Ishikawa T, Sirianni R, Tang H, McDonald JG, Yuhanna IS, Thompson B, Girard L, Mineo C, Brekken RA, Umetani M, Euhus DM, Xie Y, Shaul PW (2013). 27-Hydroxycholesterol promotes cell-autonomous, ER-positive breast cancer growth. Cell reports.

[6] Gabitova L, Gorin A, Astsaturov I (2014). Molecular pathways: sterols and receptor signaling in cancer. Clinical cancer research: an official journal of the American Association for Cancer Research.

[7] Mostaghel EA, Solomon KR, Pelton K, Freeman MR, Montgomery RB (2012). Impact of circulating cholesterol levels on growth and intratumoral androgen concentration of prostate tumors. PloS one.

[8] Raccosta L, Fontana R, Maggioni D, Lanterna C, Villablanca EJ, Paniccia A, Musumeci A, Chiricozzi E, Trincavelli ML, Daniele S, Martini C, Gustafsson JA, Doglioni C, Feo SG, Leiva A, Ciampa MG (2013). The oxysterol-CXCR2 axis plays a key role in the recruitment of tumor-promoting neutrophils. The Journal of experimental medicine.

[9] Hryniewicz-Jankowska A, Augoff K, Biernatowska A, Podkalicka J, Sikorski AF (2014). Membrane rafts as a novel target in cancer therapy. Biochimica et biophysica acta.

[10] Zhuang L, Kim J, Adam RM, Solomon KR, Freeman MR (2005). Cholesterol targeting alters lipid raft composition and cell survival in prostate cancer cells and xenografts. The Journal of clinical investigation.

[11] Nassar ZD, Hill MM, Parton RG, Parat MO (2013). Caveola-forming proteins caveolin-1 and PTRF in prostate cancer. Nature reviews Urology.

[12] Saylor PJ, Smith MR (2013). Metabolic complications of androgen deprivation therapy for prostate cancer. The Journal of urology.

[13] Perlmutter MA, Lepor H (2007). Androgen deprivation therapy in the treatment of advanced prostate cancer. Reviews in urology.

[14] White CD, Erdemir HH, Sacks DB (2012). IQGAP1 and its binding proteins control diverse biological functions. Cellular signalling.

[15] Johnson M, Sharma M, Henderson BR (2009). IQGAP1 regulation and roles in cancer. Cellular signalling.

[16] Jameson KL, Mazur PK, Zehnder AM, Zhang J, Zarnegar B, Sage J, Khavari PA (2013). IQGAP1 scaffold-kinase interaction blockade selectively targets RAS-MAP kinase-driven tumors. Nature medicine.

[17] Jenkins DE, Oei Y, Hornig YS, Yu SF, Dusich J, Purchio T, Contag PR (2003). Bioluminescent imaging (BLI) to improve and refine traditional murine models of tumor growth and metastasis. Clinical & experimental metastasis.

[18] Lin KT, Gong J, Li CF, Jang TH, Chen WL, Chen HJ, Wang LH (2012). Vav3-rac1 signaling regulates prostate cancer metastasis with elevated Vav3 expression correlating with prostate cancer progression and posttreatment recurrence. Cancer Research.

[19] Moon H, Lee CS, Inder KL, Sharma S, Choi E, Black DM, Le Cao KA, Winterford C, Coward JI, Ling MT, the Australian Prostate Cancer B, Craik DJ, Parton RG, Russell PJ, Hill MM (2013). PTRF/cavin-1 neutralizes non-caveolar caveolin-1 microdomains in prostate cancer. Oncogene.

[20] Sturge J, Caley MP, Waxman J (2011). Bone metastasis in prostate cancer: emerging therapeutic strategies. Nature reviews Clinical oncology.

[21] Siegel R, Ma J, Zou Z, Jemal A (2014). Cancer statistics, 2014. CA: a cancer journal for clinicians.

[22] Solomon KR, Pelton K, Boucher K, Joo J, Tully C, Zurakowski D, Schaffner CP, Kim J, Freeman MR (2009). Ezetimibe is an inhibitor of tumor angiogenesis. The American journal of pathology.

[23] Feng Y, Zhu Y, Chen X, Sha J, Fan L, Chen Q (2005). Effects of diet-induced hypercholesterolemia on testosterone-regulated protein expression in mice liver. Journal of nanoscience and nanotechnology.

[24] Martinez-Martos JM, Arrazola M, Mayas MD, Carrera-Gonzalez MP, Garcia MJ, Ramirez-Exposito MJ (2011). Diet-induced hypercholesterolemia impaired testicular steroidogenesis in mice through the renin-angiotensin system. General and comparative endocrinology.

[25] Inder KL, Zheng YZ, Davis MJ, Moon H, Loo D, Nguyen H, Clements JA, Parton RG, Foster LJ, Hill MM (2012). Expression of PTRF in PC-3 Cells modulates cholesterol dynamics and the actin cytoskeleton impacting secretion pathways. Molecular & cellular proteomics: MCP.

[26] Nguyen HD, Wood IA, Hill MM (2012). A robust permutation test for quantitative SILAC proteomics experiments. J Integr OMICS.

[27] Chen D, Shah A, Nguyen H, Loo D, Inder KL, Hill MM (2014). Online Quantitative Proteomics p-value Calculator for Permutation-Based Statistical Testing of Peptide Ratios. Journal of proteome research.

[28] White CD, Brown MD, Sacks DB (2009). IQGAPs in cancer: a family of scaffold proteins underlying tumorigenesis. FEBS letters.

[29] Tahir SA, Kurosaka S, Tanimoto R, Goltsov AA, Park S, Thompson TC (2013). Serum caveolin-1, a biomarker of drug response and therapeutic target in prostate cancer models. Cancer biology & therapy.

[30] Parton RG, del Pozo MA (2013). Caveolae as plasma membrane sensors, protectors and organizers. Nature reviews Molecular cell biology.

[31] Vetterkind S, Poythress RH, Lin QQ, Morgan KG (2013). Hierarchical scaffolding of an ERK1/2 activation pathway. Cell communication and signaling: CCS.

[32] Wickstrom SA, Lange A, Hess MW, Polleux J, Spatz JP, Kruger M, Pfaller K, Lambacher A, Bloch W, Mann M, Huber LA, Fassler R (2010). Integrin-linked kinase controls microtubule dynamics required for plasma membrane targeting of caveolae. Developmental cell.

[33] Lingwood D, Simons K (2007). Detergent resistance as a tool in membrane research. Nature protocols.

[34] Ariotti N, Fernandez-Rojo MA, Zhou Y, Hill MM, Rodkey TL, Inder KL, Tanner LB, Wenk MR, Hancock JF, Parton RG (2014). Caveolae regulate the nanoscale organization of the plasma membrane to remotely control Ras signaling. The Journal of cell biology.

[35] Sugie S, Mukai S, Tsukino H, Toda Y, Yamauchi T, Nishikata I, Kuroda Y, Morishita K, Kamoto T (2013). Increased plasma caveolin-1 levels are associated with progression of prostate cancer among Japanese men. Anticancer research.

[36] Gumulec J, Sochor J, Hlavna M, Sztalmachova M, Krizkova S, Babula P, Hrabec R, Rovny A, Adam V, Eckschlager T, Kizek R, Masarik M (2012). Caveolin-1 as a potential high-risk prostate cancer biomarker. Oncology reports.

[37] Hill MM, Bastiani M, Luetterforst R, Kirkham M, Kirkham A, Nixon SJ, Walser P, Abankwa D, Oorschot VM, Martin S, Hancock JF, Parton RG (2008). PTRF-Cavin, a conserved cytoplasmic protein required for caveola formation and function. Cell.

[38] Dong P, Nabeshima K, Nishimura N, Kawakami T, Hachisuga T, Kawarabayashi T, Iwasaki H (2006). Overexpression and diffuse expression pattern of IQGAP1 at invasion fronts are independent prognostic parameters in ovarian carcinomas. Cancer letters.

[39] Chen F, Zhu HH, Zhou LF, Wu SS, Wang J, Chen Z (2010). IQGAP1 is overexpressed in hepatocellular carcinoma and promotes cell proliferation by Akt activation. Experimental & molecular medicine.

[40] Hayashi H, Nabeshima K, Aoki M, Hamasaki M, Enatsu S, Yamauchi Y, Yamashita Y, Iwasaki H (2010). Overexpression of IQGAP1 in advanced colorectal cancer correlates with poor prognosis-critical role in tumor invasion. International journal of cancer Journal international du cancer.

[41] Liu Z, Liu D, Bojdani E, El-Naggar AK, Vasko V, Xing M (2010). IQGAP1 plays an important role in the invasiveness of thyroid cancer. Clinical cancer research: an official journal of the American Association for Cancer Research.

[42] Wang XX, Li XZ, Zhai LQ, Liu ZR, Chen XJ, Pei Y (2013). Overexpression of IQGAP1 in human pancreatic cancer. Hepatobiliary & pancreatic diseases international: HBPD INT.

[43] Shah A, Chen D, Boda AR, Foster LJ, Davis MJ, Hill MM (2015). RaftProt: mammalian lipid raft proteome database. Nucleic acids research.

[44] Poston CN, Duong E, Cao Y, Bazemore-Walker CR (2011). Proteomic analysis of lipid raft-enriched membranes isolated from internal organelles. Biochemical and biophysical research communications.

[45] Arielly SS, Ariel M, Yehuda R, Scigelova M, Yehezkel G, Khalaila I (2012). Quantitative analysis of caveolin-rich lipid raft proteins from primary and metastatic colorectal cancer clones. Journal of proteomics.

[46] Lin SL, Chien CW, Han CL, Chen ES, Kao SH, Chen YJ, Liao F (2010). Temporal proteomics profiling of lipid rafts in CCR6-activated T cells reveals the integration of actin cytoskeleton dynamics. Journal of proteome research.

[47] Solstad T, Bjorgo E, Koehler CJ, Strozynski M, Torgersen KM, Tasken K, Thiede B (2010). Quantitative proteome analysis of detergent-resistant membranes identifies the differential regulation of protein kinase C isoforms in apoptotic T cells. Proteomics.

[48] Foster LJ, De Hoog CL, Mann M (2003). Unbiased quantitative proteomics of lipid rafts reveals high specificity for signaling factors. Proceedings of the National Academy of Sciences of the United States of America.

[49] Babina IS, McSherry EA, Donatello S, Hill AD, Hopkins AM (2014). A novel mechanism of regulating breast cancer cell migration via palmitoylation-dependent alterations in the lipid raft affiliation of CD44. Breast cancer research: BCR.

[50] Raghu H, Sodadasu PK, Malla RR, Gondi CS, Estes N, Rao JS (2010). Localization of uPAR and MMP-9 in lipid rafts is critical for migration, invasion and angiogenesis in human breast cancer cells. BMC cancer.

[51] Luk SU, Yap WN, Chiu YT, Lee DT, Ma S, Lee TK, Vasireddy RS, Wong YC, Ching YP, Nelson C, Yap YL, Ling MT (2011). Gamma-tocotrienol as an effective agent in targeting prostate cancer stem cell-like population. International journal of cancer Journal international du cancer.

[52] Nassar ZD, Moon H, Duong T, Neo L, Hill MM, Francois M, Patron RG, Parat MO (2013). PTRF/Cavin-1 decreases prostate cancer angiogenesis and lymphangiogenesis. Oncotarget.

[53] Inder KL, Loo D, Zheng YZ, Parton RG, Foster LJ, Hill MM (2012). Normalization of protein at different stages in SILAC subcellular proteomics affects functional analysis. J Integr OMICS.

[54] Aung CS, Hill MM, Bastiani M, Parton RG, Parat MO (2011). PTRF-cavin-1 expression decreases the migration of PC3 prostate cancer cells: role of matrix metalloprotease 9. European journal of cell biology.

[55] Kielar D, Dietmaier W, Langmann T, Aslanidis C, Probst M, Naruszewicz M, Schmitz G (2001). Rapid quantification of human ABCA1 mRNA in various cell types and tissues by real-time reverse transcription-PCR. Clinical chemistry.

[56] Wong J, Quinn CM, Brown AJ (2007). Synthesis of the oxysterol, 24(S), 25-epoxycholesterol, parallels cholesterol production and may protect against cellular accumulation of newly-synthesized cholesterol. Lipids in health and disease.

