# Supplementary material for: Diet-induced hypercholesterolemia promotes androgen-independent prostate cancer metastasis via IQGAP1 and caveolin-1
Source: Oncotarget. 2015 Mar 2;6(10):7438–53. doi: 10.18632/oncotarget.3476 (PMC4480691; doi:10.18632/oncotarget.3476)
Supplement: Supplementary file 1 [file oncotarget-06-7438-s001.pdf]

Diet-induced hypercholesterolemia promotes androgen-independent prostate cancer metastasis via IQGAP1 and caveolin-1

Supplementary Material

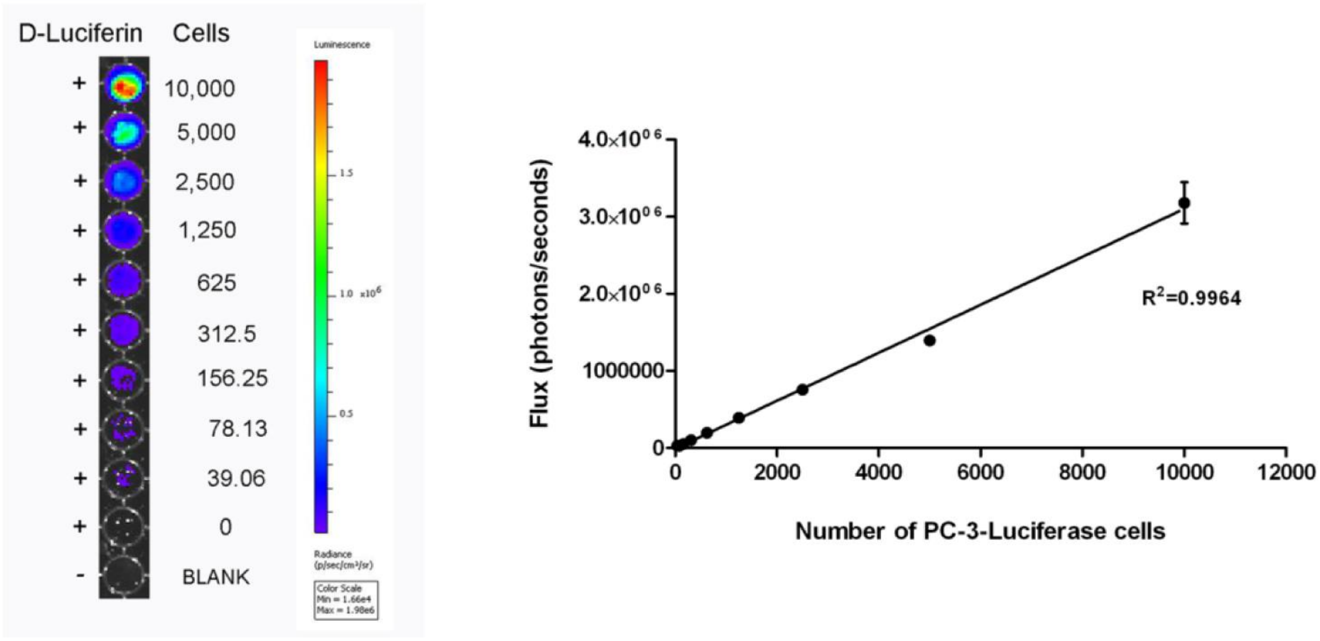

Supplementary Figure S1: *In vitro* bioluminescence of PC-3-luciferase cells measured by the IVIS spectrum. Serial dilution was performed from 10,000 cells in a black 96-well plate following by 5 hours incubation in a tissue culture incubator. After adding D-luciferin, *in vitro* bioluminescence was determined using IVIS spectrum, and the intensity was quantified using Live imaging software. Linear regression, $R^2 = 0.9964$, $n = 3$.

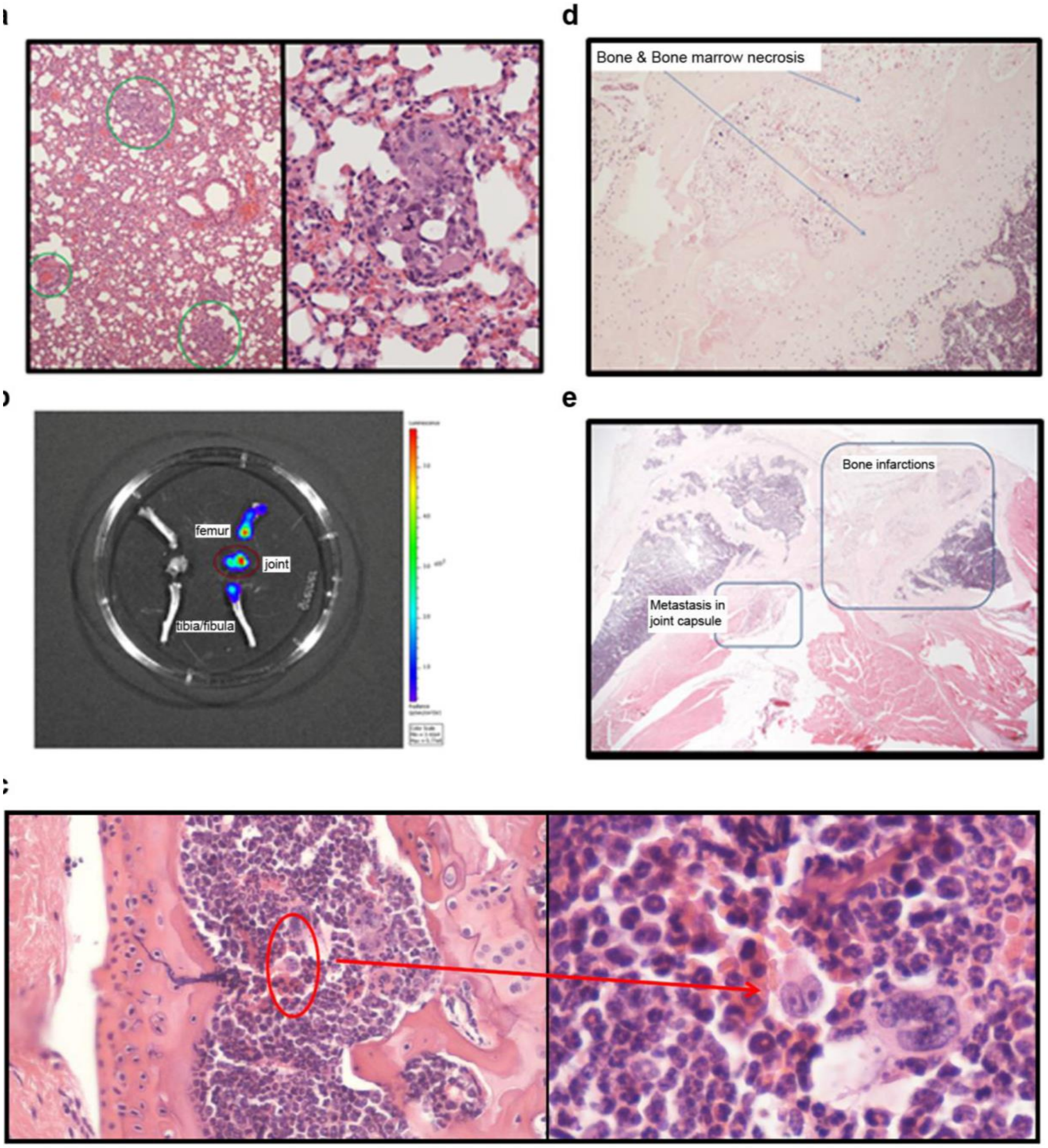

Supplementary Figure S2. Metastatic PC-3 dissemination to the lung and bones including joint tissues. (a) Histopathology revealed microscopic lung metastases with many, randomly scattered, cell-rich micrometastases in all lobes (green circles). Infiltrating inflammatory cells (polymorphonuclear leukocytes and macrophages) were also visible. (b) *Ex vivo* imaging demonstrated bone metastasis but also showed strong positive bioluminescence at the joint. (c) Histopathology revealed microscopic bone metastases. Representative figure shows a single cluster of 2-3 PC-3 cells in the bone marrow of tibia (arrow). (d) Bone and bone marrow necrosis were observed possibly due to tumor invasion through either blood vessels and/or lymphatic vessels. (e) Histopathology revealed pathological bone infarction and metastases in joint capsules.

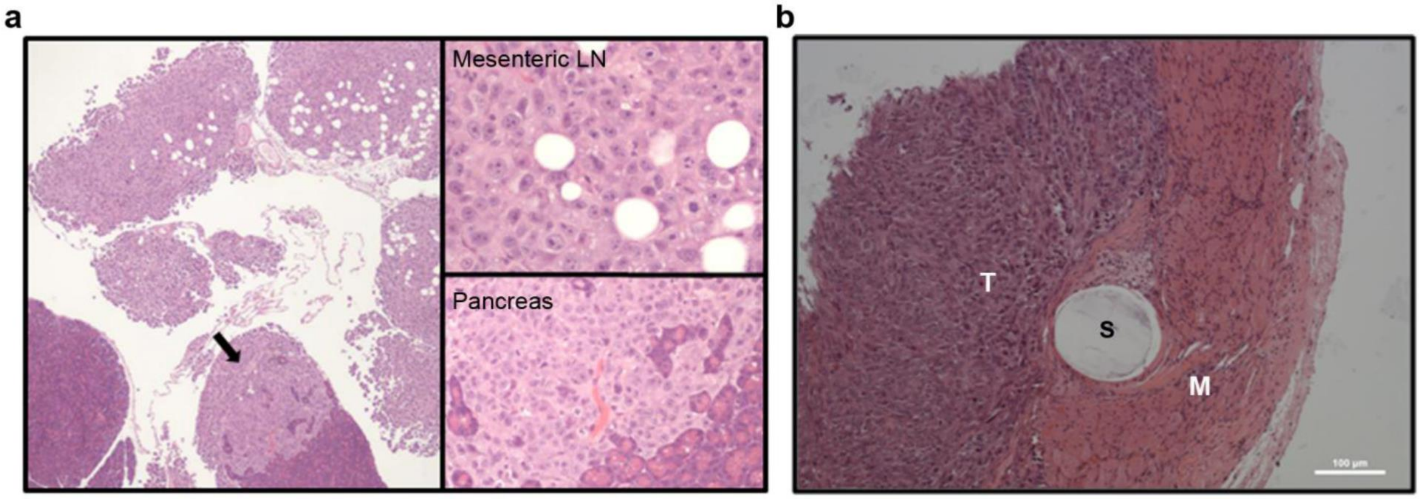

Supplementary Figure S3. Tumor cell invasion to surrounding tissues. (a) Histopathology on lymph node metastases revealed tumor cells invading into the pancreas in regions in direct contact with the mesenteric lymph node tumors (arrow; tumor invasion to the pancreas, LN; lymph node). (b) Tumor cells invading into abdominal muscular layers due to the contact of prostate tumors (T, prostate tumor; S, surgical suture; M, muscular layer; Bar, 100 μm).

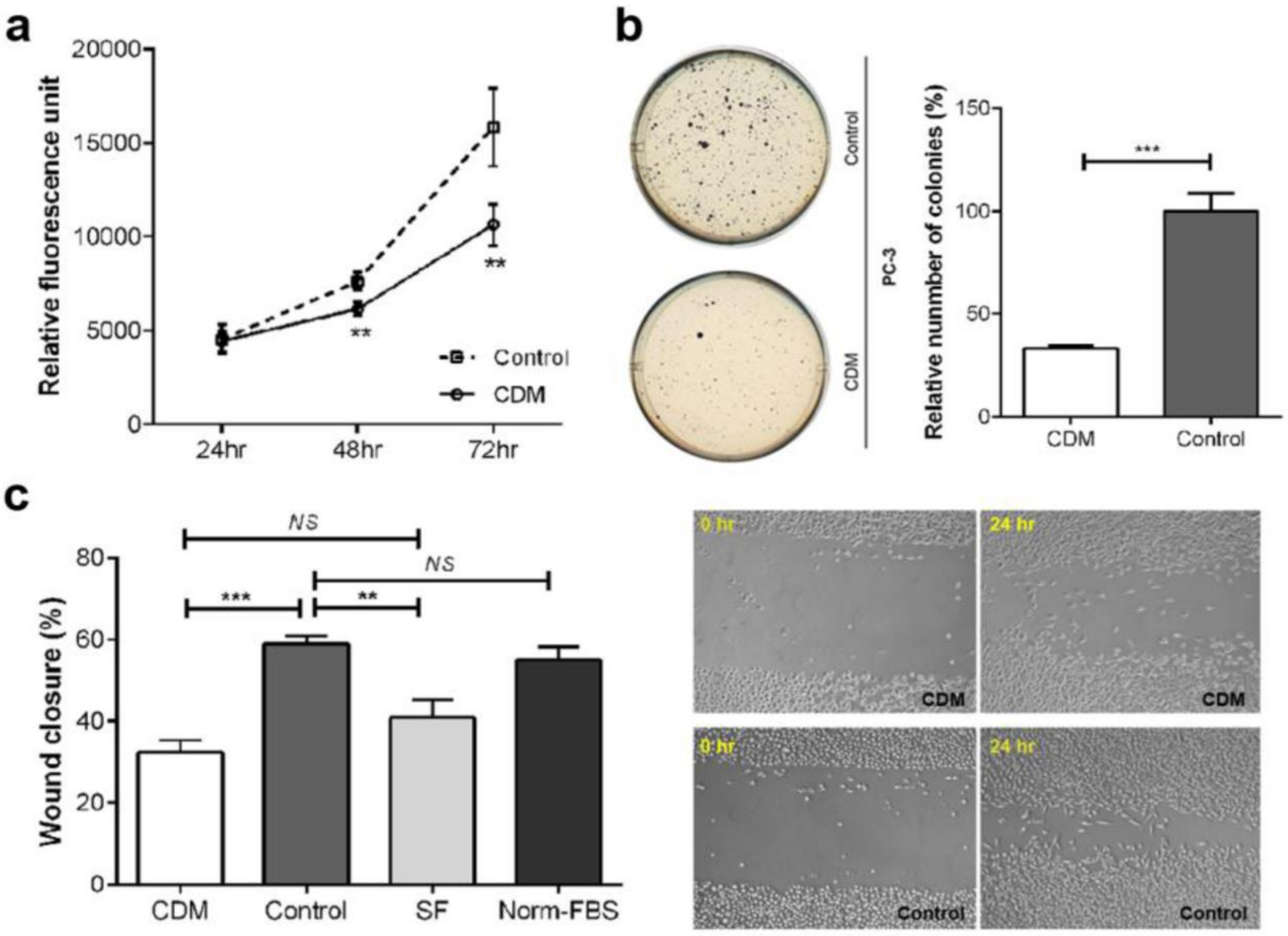

Supplementary Figure S4. Cholesterol-deficient medium reduced PC-3 proliferation, anchorage- independent growth and migration. (a) PC-3 cells plated in black 96-well plates were cultured in media containing 10% delipidated FBS (CDM) or control dialyzed FBS (Control). Cell proliferation was determined at 24, 48 and 72 hours using Alamar Blue assay (mean ± SEM, $n = 3$, $**p < 0.005$). (b) To assess anchorage-independent growth, colony formation in soft agar was performed by plating 1 x 10^4 PC-3 cells in 6 well plates between 0.35% agar and 0.5% agar. Viable colonies were stained with 3-(4,5-Dimethylthiazol-2-yl)-2,5-Diphenyltetrazolium Bromide (MTT, Sigma-Aldrich) after 21 days. The relative number of colonies was compared between CDM and control (mean ± SEM, $n = 4$, $***p < 0.0005$). (c) Migration was measured in a wound healing assay using CDM, control media serum free (SF) or medium containing 10% normal FBS (Norm-FBS). For wound healing assays, PC-3 cells were plated in 6 well plates and grown to 95% confluence. After wounding the monolayer using micropipette tips, cells were gently washed 3 times with PBS, and the growth medium was replaced. Three to four different wound areas per well were monitored by a Live cell imager (Zeiss axioimager live cell system using axiovision software) every 30 minutes, and the percent of 24 hour wound closure was calculated as % wound closure = $[(W_{0hr}-W_{24hr})/W_{0hr}] \times 100$. Mean ± SEM, $n = 4$ for CDM and control, $n = 3$ for SF and Norm-FBS, $**p < 0.005$, $***p < 0.0005$. Similar to serum free media, cholesterol-deficiency significantly reduced PC-3 migration indicating the potential role of cholesterol in migration.

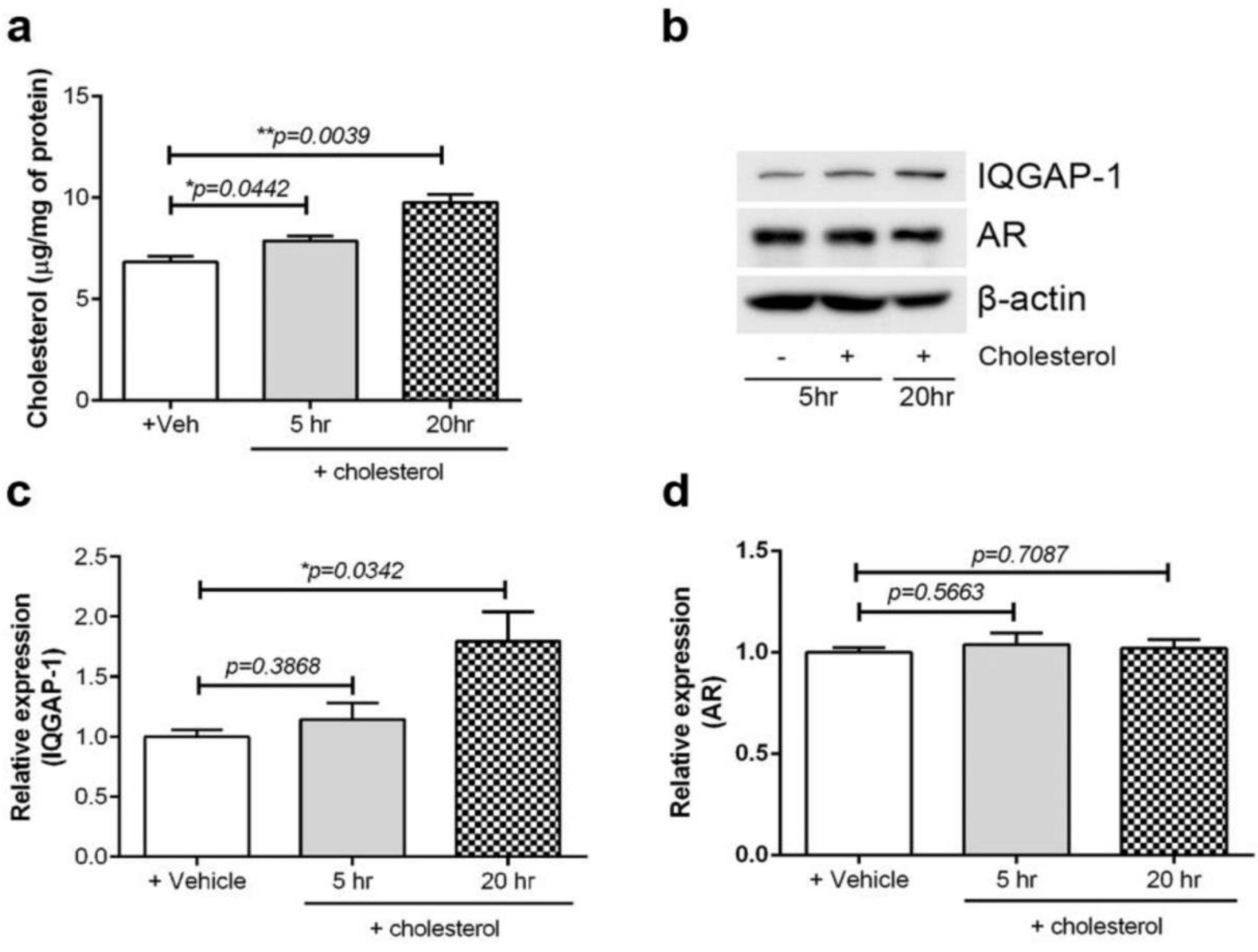

Supplementary Figure S5. Effect of cholesterol treatment on IQGAP1 and androgen receptor protein levels in LNCaP cells. LNCaP cells were incubated with 5 μM cholesterol or vehicle for 5 hours or 20 hours. (a) Total lysates were collected from LNCaP cells after cholesterol treatment, and total cellular cholesterol levels were measured (mean ± SEM, $*p < 0.05$, $**p < 0.005$). (b) Cell lysates (20 μg) were separated using SDS-PAGE, and immunoblotted with IQGAP1, androgen receptor (AR) and β-actin as indicated. Relative expression of (c) IQGAP1 and (d) androgen receptor was quantified using Image J, and values are represented in bar graphs (mean ± SEM, $*p < 0.05$).

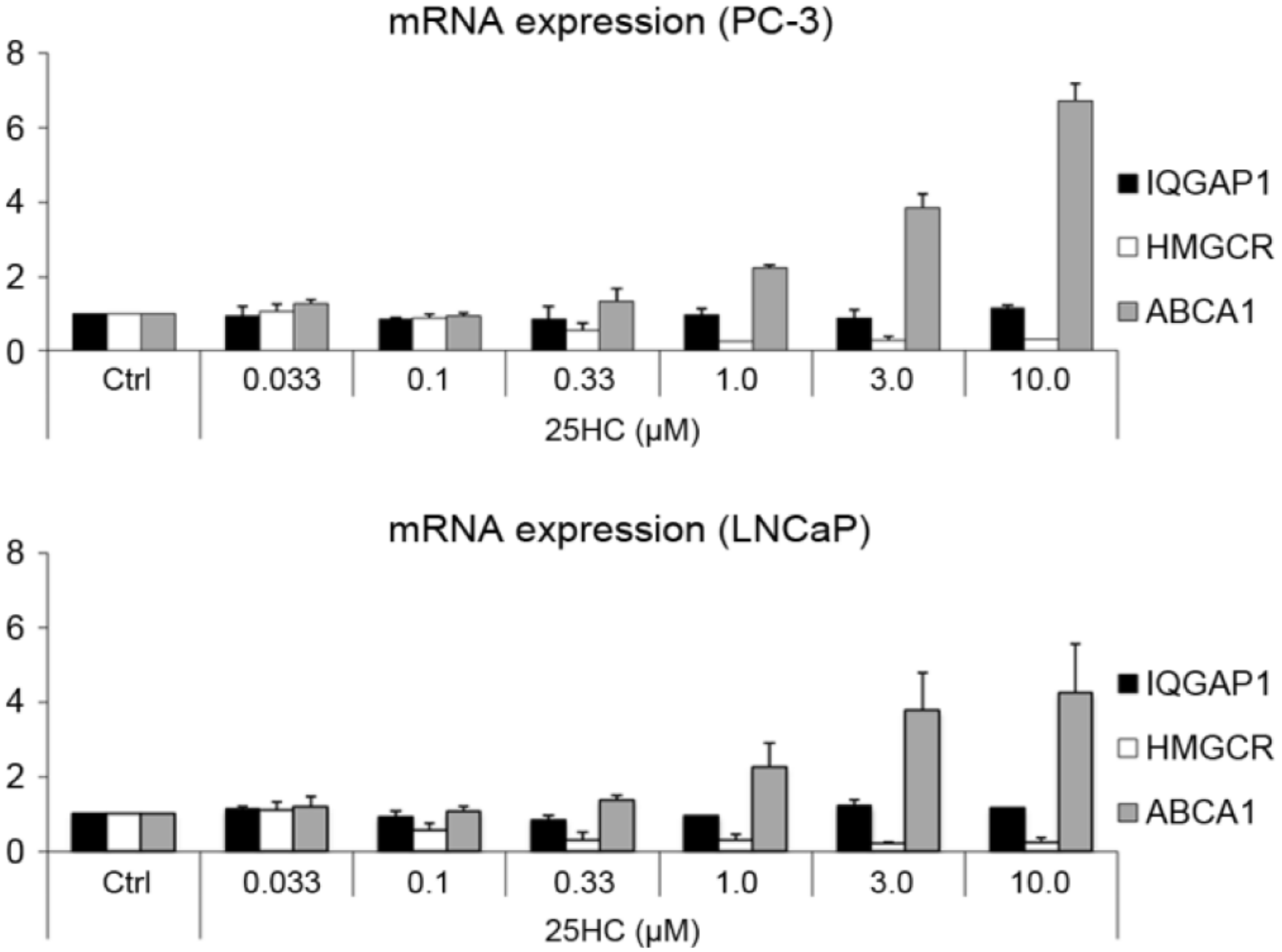

Supplementary Figure S6. mRNA expression of IQGAP1, HMGCR and ABCA1 in PC-3 and LNCaP cells. PC-3 and LNCaP cells were pretreated in low cholesterol medium overnight, and then treated for 6 hours with the indicated concentration of 25-hydroxycholesterol. RNA was harvested and reverse transcribed to cDNA. mRNA levels of IQGAP1, HMGCR, and ABCA1 were measured and normalized to PBGD. Data presented as mean ± SEM from two separate experiments, each performed in triplicate.

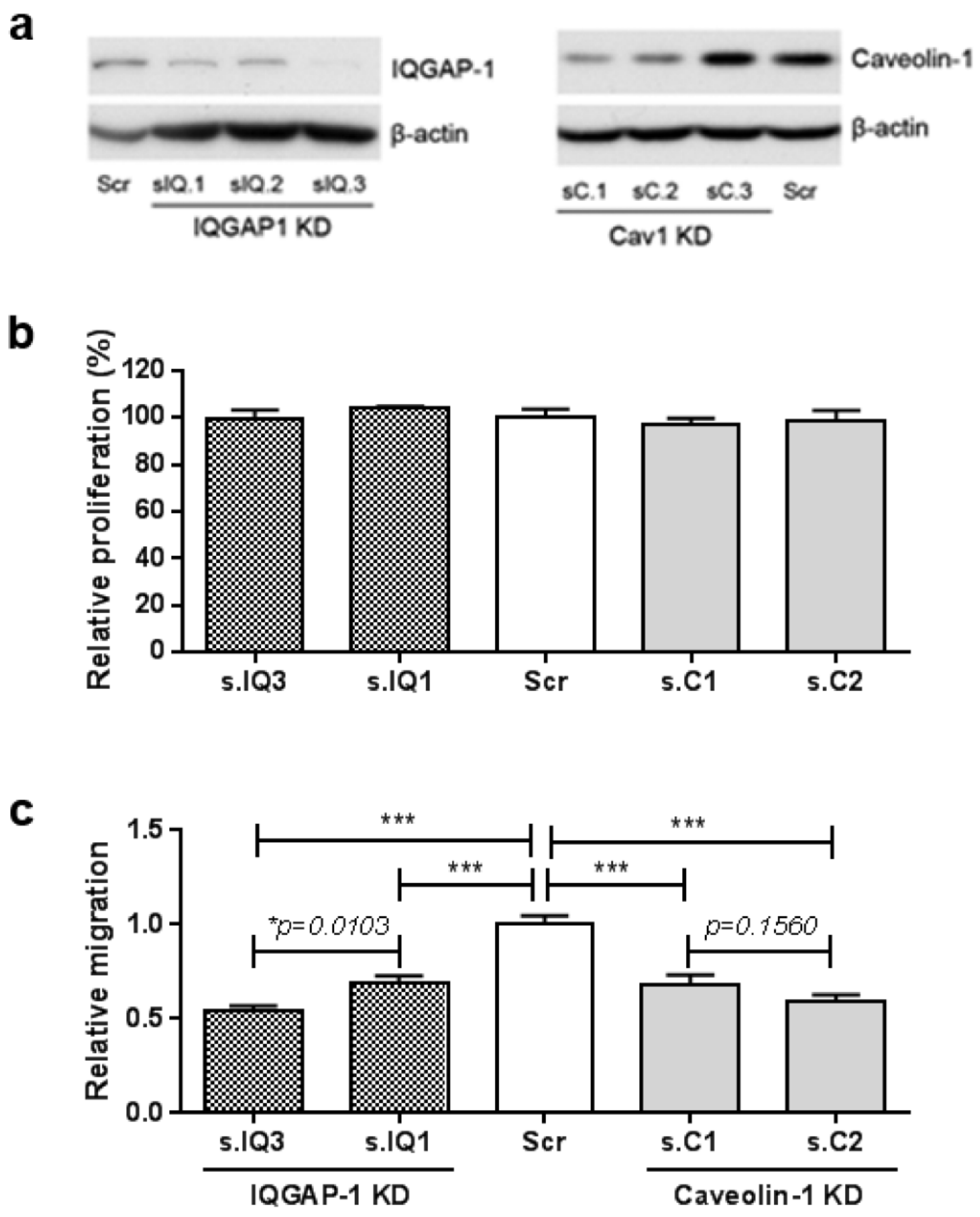

Supplementary Figure S7. Knockdown of IQGAP1 or caveolin-1 using shRNA, and *in vitro* functional assays. (a) IQGAP1 or caveolin-1 knockdown of PC-3 cells were generated using lentiviral mediated shRNAs and expression examined by immunoblotting, after sorting pure populations by flow cytometry. The first and third IQGAP1 shRNAs (sIQ1 and sIQ3), and the first and second caveolin-1 shRNAs (sC1 and sC2) showed the best efficiency in the knockdown of IQGAP1 or caveolin-1, and were selected for *in vitro* functional characterisation measuring proliferation (b) and transmigration (c) as described in Materials and Methods. (n=3, mean ± SEM, $*p < 0.05$, $***p < 0.0005$)

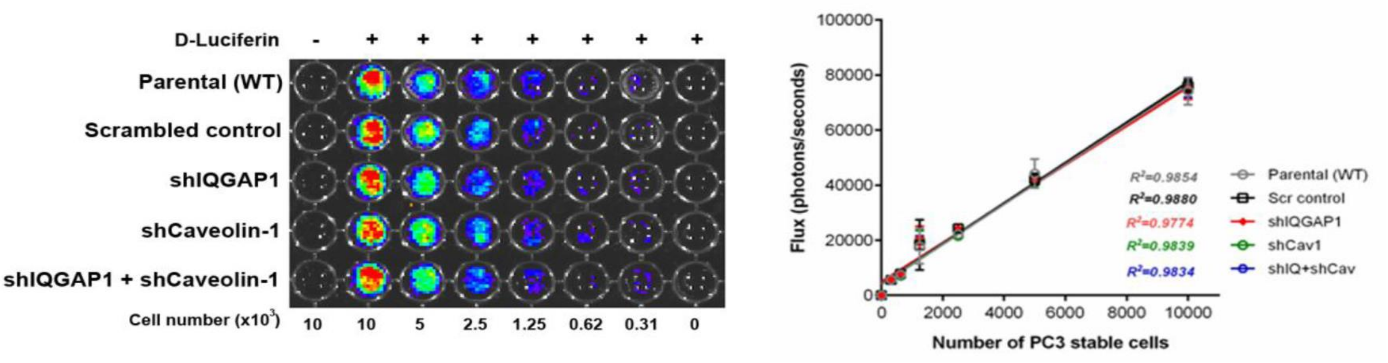

Supplementary Figure S8. *In vitro* bioluminescence of PC-3-luciferase shRNA cell lines. PC3- luciferase cell lines with different shRNA were plated in black 96 well plates and bioluminescence measured after adding D-luciferin. Similar *in vitro* bioluminescence intensity and linearity of bioluminescence as cell numbers increased: Parental (R^2 = 0.9854), scrambled control (R^2 = 0.9880), shIQGAP1 (R^2 = 0.9774), shcaveolin-1 (R^2 = 0.9839) and double knockdown of IQGAP1 and caveolin-1 (R^2 = 0.9834).

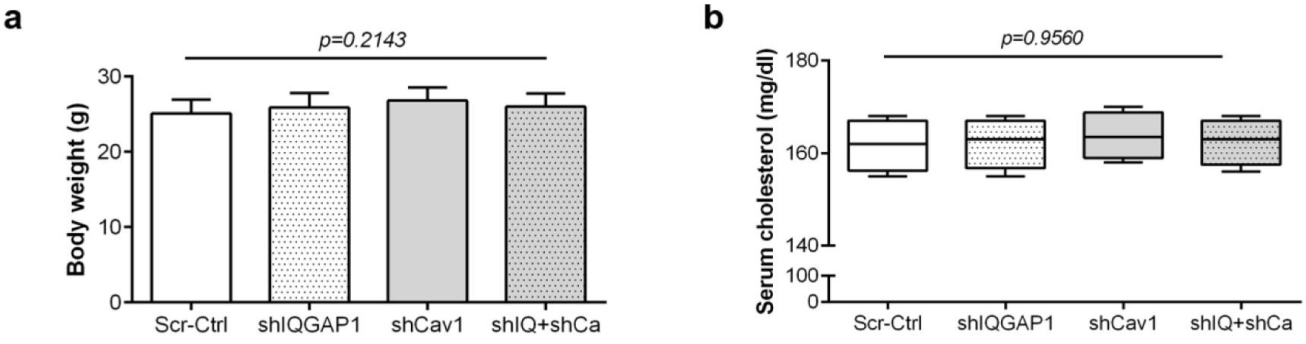

Supplementary Figure S9. Clinical parameters of control, and IQGAP1 and/or caveolin-1 knockdown groups. (a) Body weight and (b) serum cholesterol levels of mice with xenografted shRNA PC3-luciferase cells. shRNA PC3-luciferase cells were injected in to the prostate of mice on a hypercholesterolemic diet. The body weight and serum cholesterol levels between mice of different groups were similar after growth of the xenografts for 6 weeks.
